# Microfluidic Biosensors for the Detection of Foodborne Pathogens: A Comprehensive Review

**DOI:** 10.1002/fsn3.72588

**Published:** 2026-11-09

**Authors:** Wanjiao Mao, Li Zhang

**Affiliations:** ^1^ Southwest Medical University Luzhou Sichuan China; ^2^ Health Management Center The Affiliated Hospital, Southwest Medical University Luzhou Sichuan China

**Keywords:** biofouling, CRISPR‐Cas Systems, food safety, lab‐on‐a‐chip devices, point‐of‐care systems

## Abstract

Foodborne pathogen detection in complex foods remains challenging because sample preparation and signal readout can be affected by lipids, proteins, and background microorganisms. This review compares microfluidic biosensors by substrate, fabrication route, sample handling, and optical, electrochemical, or nucleic‐acid readout. Published studies demonstrate rapid detection in selected food samples, but reported detection limits cannot be compared without their matrices, units, enrichment steps, and assay times. Laboratory performance also does not establish field readiness. Reproducible manufacture, reagent stability, integrated sample‐to‐answer operation, and validation in authentic foods remain major translational barriers. Orthogonal readouts, CRISPR‐assisted assays, and AI‐supported interpretation are promising, but their benefit over simpler methods requires direct comparison and independent validation. Future studies should report matrix‐specific recovery and performance, test robustness across sites, and address usability, cost, and regulatory requirements before claiming commercial readiness.

## Graphical abstract

Microfluidic biosensors offer several approaches to detecting foodborne pathogens in meat, milk, and fresh produce. The schematic shows food samples entering a lab‐on‐a‐chip platform, where microchannels direct them toward sensing regions. Pathogen‐associated signals can then be measured through alternative colorimetric, fluorescence, or digital readouts. As discussed in this review, translating these approaches to routine food testing requires attention to sample preparation, interference from complex food matrices, and validation in authentic samples.

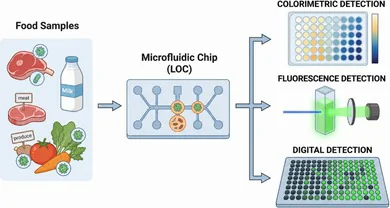

## 1 Introduction

Foodborne pathogens represent a pervasive global health challenge, imposing a substantial burden on public health infrastructures and socio‐economic stability. According to official data from the World Health Organization (WHO), contaminated food results in an estimated 600 million illnesses and 420,000 deaths globally each year. The magnitude and complexity of this burden are frequently highlighted in authoritative journals; for example, the escalating threat of antimicrobial resistance among these pathogens has been extensively reviewed (Ikhimiukor et al. 2022), while their global mortality impacts are rigorously tracked (Marchello et al. 2022). The clinical manifestations of these illnesses vary significantly depending on the etiological agent and its mechanism of pathogenicity. For instance, *Salmonella* infection typically presents as acute gastroenteritis characterized by fever, diarrhea, and abdominal cramps (Galán 2021). In contrast, invasive listeriosis, caused by the psychrotrophic bacterium *Listeria monocytogenes* , disproportionately affects vulnerable groups and can progress to life‐threatening complications such as meningitis and septicemia. Furthermore, foodborne illness may arise from intoxication rather than infection; notably, the ingestion of thermostable enterotoxins produced by *Staphylococcus aureus* induces a rapid‐onset intoxication, predominantly manifesting as severe nausea and vomiting (Mairi et al. 2025).

Despite the urgent need for effective monitoring, traditional detection methodologies face significant limitations (Panwar et al. 2023). Conventional culture‐based techniques, long considered the “gold standard,” are inherently labor‐intensive and time‐consuming, typically requiring 3 to 5 days for preliminary identification and biochemical confirmation (Elbehiry et al. 2023; Sogore et al. 2024). Furthermore, these methods often fail to detect pathogens in the VBNC state, potentially leading to false‐negative results (Zhang et al. 2023). While alternative molecular and immunological approaches—such as Polymerase Chain Reaction (PCR) and Enzyme‐Linked Immunosorbent Assay (ELISA)—offer improved specificity, they are not without drawbacks (Li et al. 2022; Zhan et al. 2024). PCR‐based methods generally require complex nucleic acid extraction, expensive thermal cycling equipment, and highly trained personnel, which limits their utility for on‐site testing. Similarly, conventional biosensing often struggles with matrix interference in complex food samples (Quintela et al. 2022). These constraints highlight a critical gap in current food safety assurance systems: the need for a detection platform that combines the sensitivity of molecular methods with the speed, portability, and cost‐effectiveness required for field application (Yadav and Yadav 2025).

Microfluidics has emerged as a transformative technology capable of bridging this gap. Briefly defined as fluid manipulation at the sub‐millimeter scale to enhance reaction kinetics, microfluidics directly addresses the severe analytical challenges posed by complex food matrices. Unlike traditional discrete methods, it enables the seamless on‐chip integration of target enrichment, nucleic acid extraction, and downstream detection, effectively bypassing laborious off‐chip pretreatment (Cheng et al. 2025; Mairi et al. 2025). Operating with an exceptionally low sample volume, these “Lab‐on‐a‐Chip” (LOC) platforms offer miniaturized, automated analysis (Ma et al. 2024). Fabricated from diverse materials ranging from traditional polymers to emerging paper‐based substrates, microfluidic devices exhibit high compatibility with portable readout systems, such as smartphone‐based optical or electrochemical sensors (Abdelhamid et al. 2025; Sen et al. 2024). Consequently, this integration allows for precise microenvironmental control, unlocking the immense potential for the field deployment of rapid foodborne pathogen detection (Diep Trinh et al. 2024).

Microfluidic platforms have achieved widespread adoption within the scientific community, primarily due to the distinct advantages they offer over traditional macro‐scale systems (Jiang et al. 2024). These systems encompass a diverse array of functionalities and significantly enhance the efficiency of intricate laboratory workflows (Kapoor et al. 2024). A fundamental technical advantage of microfluidic chips is their capacity for the precise manipulation of minute fluid volumes at the microscale, which facilitates the seamless integration of complex biochemical assays and sample processing steps (Li et al. 2023). Furthermore, microfluidic chips offer several critical benefits, including reduced analytical duration, minimal reagent consumption, and the capacity for high‐throughput multiplexing (Lee and Kim 2024). The miniaturized dimensions of these devices accelerate reaction kinetics by substantially increasing the surface‐area‐to‐volume ratio (Squires and Quake 2005). Consequently, these platforms have emerged as a pivotal technology specifically tailored for food safety monitoring, enabling the rapid and sensitive on‐site detection of foodborne pathogens, toxins, and chemical contaminants (Diep Trinh et al. 2024).

Despite these extensive foundational developments, a critical analysis of the contemporary academic landscape reveals that the literature concerning microfluidic biosensors for foodborne pathogen detection has become highly saturated. Recent reviews published between 2023 and 2026 have comprehensively documented the historical transition from culture‐based methods to rapid microfluidic screening, exhaustively summarizing the deployment of colorimetric, fluorescent, and chemiluminescent detection paradigms alongside the integration of advanced nanomaterials. Consequently, simply enumerating recent publications without introducing a unique theoretical framework yields redundant insights.

Existing reviews describe the mechanisms and applications of microfluidic sensors, but the evidence needed for routine food testing remains uneven. This review critically discusses the translational readiness of selected microfluidic biosensing technologies without assigning a numerical maturity score to entire technology classes. It compares demonstrated analytical performance with the conditions under which it was measured, including food‐matrix effects, sample preparation, reagent stability, manufacturing, and field validation. Orthogonal recognition and multimodal readout are discussed as possible approaches to interference control; their value requires testing against suitable single‐mode comparators in authentic foods. This evidence‐based perspective identifies the studies and validation steps needed to move beyond laboratory prototypes.

## 2 Common Foodborne Harmful Microorganisms

Microbial hazards within food systems are principally classified into three categories: bacteria, viruses, and fungi. Significant bacterial pathogens include *Escherichia coli* , *Listeria monocytogenes* , *Staphylococcus aureus* , and species within the genera *Salmonella* and *Campylobacter*, all of which are established etiologies of foodborne disease. Viral agents of concern encompass Norovirus, a primary cause of gastroenteritis, alongside zoonotic pathogens such as Avian and Swine Influenza viruses that present contextual risks (As shown in Figure 1). Additionally, toxigenic fungi, notably Aspergillus flavus, Monascus purpureus, and Penicillium spp., compromise food safety primarily through the production of mycotoxins. Exposure to these agents can result in severe pathology and significant morbidity or mortality. The physicochemical complexity of the matrices harboring these microorganisms—ranging from highly viscous dairy emulsions to protein‐dense meat exudates—creates an aggressive biofouling environment that actively suppresses the Technology Readiness Level (TRL) progression of emerging biosensors, preventing their translation from pristine laboratory buffers to authentic field deployments, which can cause matrix interference and surface fouling that must be evaluated in authentic foods(Flores‐Ramírez et al. 2024).

**FIGURE 1:**
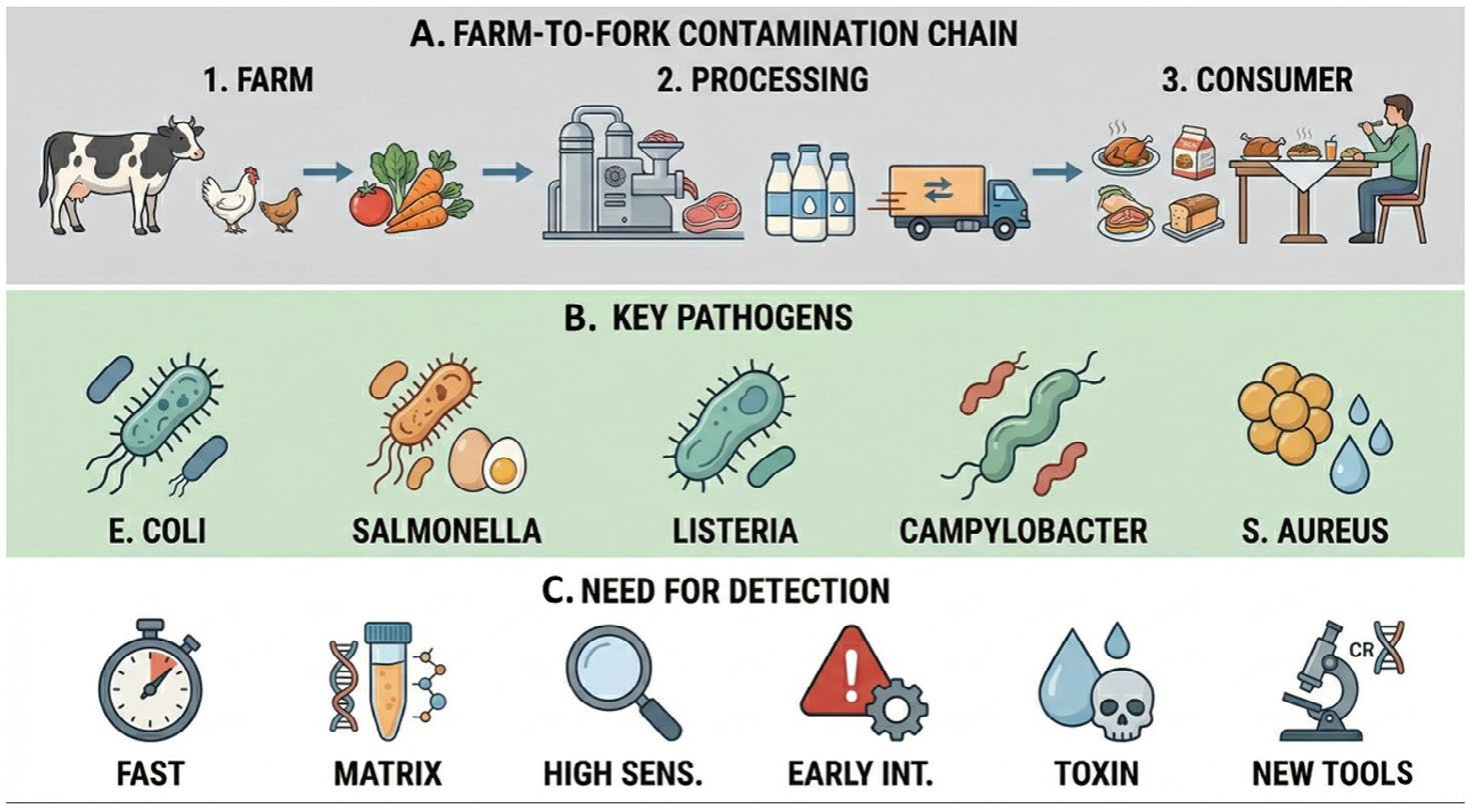
Outline of contamination chain, main pathogens and requirements of detection. (A) The chain of Farm‐to‐Fork. (B) Five types of key food pathogens. (C) The requirement of detection methods. (The initial conceptual layout and structural design of this theoretical flowchart were generated with the assistance of Nano Banana. The figure was subsequently subjected to rigorous scientific review, manual structural editing, and fact‐checking by the authors to ensure representational accuracy prior to final submission. No third‐party copyrighted images, icons, templates, or other protected visual materials were used.)

### 2.1 Pathogenic *Escherichia coli* and Global Health Burden

Pathogenic variants of *Escherichia coli* constitute a critical etiology of foodborne morbidity worldwide. Among these, Shiga toxin‐producing *E. coli* (STEC) and Enterotoxigenic *E. coli* (ETEC) represent predominant public health threats. Diagnostic differentiation relies on specific virulence targets: STEC is identified by the presence of stx1, stx2, and eae genes, whereas ETEC is characterized by heat‐labile (elt) and heat‐stable (est) enterotoxin genes (Croxen et al. 2013). These pathogens are primarily transmitted through complex food matrices, most notably contaminated bovine meat, unpasteurized dairy products, and fresh produce (Tam et al. 2024). From an analytical perspective, safeguarding the food supply requires overcoming profound detection challenges. Regulatory sampling plans specify analytical portions and decision criteria, whereas a sensor analytical limit of detection (LOD) describes performance under defined assay conditions; these quantities cannot be equated. Food‐matrix studies should report the sample mass, enrichment, recovery, and concentration presented to the sensor. Detecting low‐level contamination is heavily impeded by matrix interference, as lipids, proteins, and competitive background microflora inhibit sensor transduction and enzymatic amplification. Consequently, accurate pathogen quantification necessitates intensive sample pre‐treatment, often requiring enrichment or separation steps to effectively isolate target analytes from the inhibitory food matrix prior to downstream molecular detection (Y. Zhang, Ma, et al. 2025; Zhang, Zhao, et al. 2025). Deploying single‐mode biosensors in such environments frequently results in false positives due to non‐specific protein adsorption; therefore, achieving field deployability may benefit from orthogonal sensing architectures that cross‐validate physical and chemical signals simultaneously; their ability to reduce background noise in unpasteurized dairy and raw meat exudates still requires systematic validation (Cunha et al. 2025).

### 2.2 *Salmonella* spp.

Poultry and eggs are important matrices for Salmonella screening, including for Enteritidis and Typhimurium serovars (Pande et al. 2016). Culture methods can require enrichment before confirmation, whereas rapid molecular and microfluidic assays must contend with lipids, proteins, and competing microorganisms in these foods (Patel et al. 2024; Kubo et al. 2020; Liu et al. 2019). Reports of faster detection should specify whether and how enrichment, washing, or concentration was performed because those steps affect the total time to result and the concentration presented to the sensor.

Poultry homogenates can be opaque and viscous, making fluorescence‐based assays harder to transfer from controlled laboratory conditions to food‐testing workflows. Published performance should therefore be interpreted in light of sample preparation, recovery, and matrix‐specific inhibition. Separation or concentration modules may help, but their benefit requires direct testing with authentic poultry samples rather than an assumed maturity level.

### 2.3 *Listeria monocytogenes*

*Listeria monocytogenes* can persist and grow under refrigeration in some ready‐to‐eat foods, which makes sampling and matrix‐compatible detection important (Belias et al. 2024). Listeriolysin O and internalin‐associated factors contribute to pathogenesis; Pitti et al. (2025) report the occurrence of corresponding genes in sampled food isolates, not a direct test of invasion mechanisms. Analytical studies should specify recovery, enrichment, and interference in the food matrix because low starting concentrations and matrix components can increase false‐negative risk (Liu et al. 2026).

The persistent biofilm‐forming capability of *L. monocytogenes* on industrial food‐processing surfaces demands the deployment of robust, field‐deployable continuous‐flow microfluidic biosensors that are immune to surface fouling. Meeting this benchmark mandates the transition of academic prototypes to validated systems capable of long‐term environmental deployment without signal degradation, utilizing advanced anti‐biofouling surface chemistries and automated calibration algorithms (Fante and Grünberger 2026).

### 2.4 *Campylobacter jejuni*

*Campylobacter jejuni* is a leading global cause of bacterial gastroenteritis, typically manifesting as severe abdominal cramping, fever, nausea, and diarrhea, with Guillain‐Barré syndrome (GBS) representing its most severe post‐infectious sequela. Avian species—particularly commercially reared poultry—serve as the principal reservoir, though transmission is also frequently facilitated by the consumption of unpasteurized dairy products, untreated water, and cross‐contaminated produce. Although global incidence historically exceeds 96 million cases annually, mitigating this ubiquitous public health threat is severely hindered by formidable detection challenges. Traditional culture‐based diagnostics are notoriously labor‐intensive and time‐consuming due to the pathogen's fastidious nature, specifically requiring strict microaerophilic atmospheres for cultivation (Zhi et al. 2022). Furthermore, *C. jejuni* typically exists at extremely low abundances within complex food matrices, significantly complicating its efficient isolation from competing background microflora. Compounding these diagnostic hurdles, environmental stressors encountered during food processing and cold storage frequently induce the bacteria into a sublethally injured or VBNC state. In this dormant state, *C. jejuni* evades conventional culture‐dependent surveillance while fully retaining its pathogenic potential, significantly elevating the risk of false‐negative reporting (Zhang et al. 2023; Ricke et al. 2019). Consequently, overcoming these critical detection limitations is imperative for ensuring global food safety.

### 2.5 *Staphylococcus aureus*

Although vegetative cells of *Staphylococcus aureus* are highly susceptible to thermal inactivation, the pathogen's primary threat stems from its capacity to synthesize highly heat‐stable staphylococcal enterotoxins (SEs), such as the classical SEA and the emerging SEH (Cieza et al. 2024). Contamination of the food supply is predominantly driven by asymptomatic food handlers, who inadvertently transfer the bacteria from persistent colonization sites—specifically the anterior nares and epidermis—to food matrices via direct contact. Following improper post‐preparation storage at ambient temperatures, *S. aureus* undergoes rapid proliferation and enterotoxin accumulation. Consequently, the ingestion of these preformed toxins, rather than the bacterium itself, precipitates staphylococcal food poisoning (SFP), an acute intoxication clinically characterized by rapid‐onset nausea, violent emesis, and severe abdominal cramping. Because SFP is an intoxication rather than a direct bacterial infection, food safety surveillance necessitates distinguishing between two fundamentally different technical routes: pathogen detection and toxin detection (Mairi et al. 2025). While molecular pathogen detection confirms toxigenic genetic potential, it fails to verify functional toxin expression; conversely, distinct toxin detection platforms are imperative to quantify the actual preformed SEs responsible for the disease (Q. Li, Dou, et al. 2024; Li, Wang, et al. 2024). In protein‐ and lipid‐rich prepared foods, post‐processing temperature abuse can permit toxin accumulation, and analytical workflows should distinguish viable cells from preformed enterotoxins while reducing matrix‐driven signal interference (Mairi et al. 2025; Q. Li, Dou, et al. 2024; Li, Wang, et al. 2024).

### 2.6 Other Notable Pathogens

Beyond the aforementioned bacteria, the imperative for advanced detection platforms extends to diverse public health threats posed by *Bacillus* species, *Vibrio* species, noroviruses, and mycotoxigenic Aspergillus. *Bacillus cereus* remains a ubiquitous foodborne pathogen causing emetic and diarrheal syndromes, whereas *Bacillus anthracis* causes anthrax, a severe zoonosis necessitating rapid, on‐site biosensing for trace spore identification (Wang et al. 2021). Additionally, *Vibrio* species (e.g., *V. vulnificus* and *V. parahaemolyticus* ) drive critical seafood‐associated infections, with *V. vulnificus* causing approximately 95% of seafood‐related fatalities in the United States, underscoring the demand for ultrasensitive multiplexed monitoring platforms like thin‐film impedance sensors (Pérez Roig et al. 2024). Furthermore, norovirus is a primary viral etiology of epidemic gastroenteritis, accounting for approximately 20% of acute gastroenteritis cases globally (roughly 685 million infections annually). To mitigate these massive outbreaks and overcome the lack of robust in vitro cultivation, novel diagnostic platforms—such as CRISPR‐based assays and aptamer‐functionalized biosensors—have become critical for point‐of‐care viral surveillance (Marpaung et al. 2025). Finally, Aspergillus flavus poses a food‐safety risk through aflatoxin production. Xie et al. (2018) used Orbitrap mass spectrometry to profile metabolites in the aflatoxin biosynthesis pathway. Applying biosensors to aflatoxins in foods is a separate analytical question that requires matrix‐specific validation.

## 3 Types and Fabrication of Microfluidic Chips

Transitioning from the biological profiling of foodborne pathogens to the engineering of point‐of‐need (PON) diagnostics requires a critical logical bridge: the selection of microfluidic substrate materials (Ren et al. 2013). This foundational decision intrinsically dictates five key performance indicators essential for field deployment: cost‐effectiveness, optical transparency, fabrication scalability (mass production), amenability to surface modification, and device portability (Du et al. 2024). Although early microfluidic systems relied predominantly on silicon and glass inherited from the semiconductor industry, the unique demands of pathogen detection—specifically, the necessity to process heterogeneous matrices (e.g., meat homogenates, dairy) and the urgent need for low‐cost, disposable diagnostics—have catalyzed a paradigm shift toward diverse polymeric and cellulosic materials (Wang et al. 2022). Contemporary microfluidic architectures are primarily categorized into elastomers, rigid thermoplastics, inorganic substrates, and cellulosic paper (As shown in Figure 2). Because each material class exhibits inherent trade‐offs among these five key indicators, researchers must implement a rigorous “fit‐for‐purpose” selection strategy. This tailored approach ensures optimal isolation and sensing capabilities for specific biological hazards, such as *Salmonella enterica* , *Escherichia coli* , and *Listeria monocytogenes* (Sohrabi et al. 2022). Consequently, modern food monitoring increasingly relies on hybrid microfluidic systems that synergize distinct material properties to achieve fully integrated, sample‐to‐answer diagnostic functionalities. From a commercialization standpoint, fabrication methods affect manufacturing reproducibility and cost, but the readiness of a specific biosensor depends on its integrated performance, validation in authentic foods, and testing under intended operating conditions.

**FIGURE 2:**
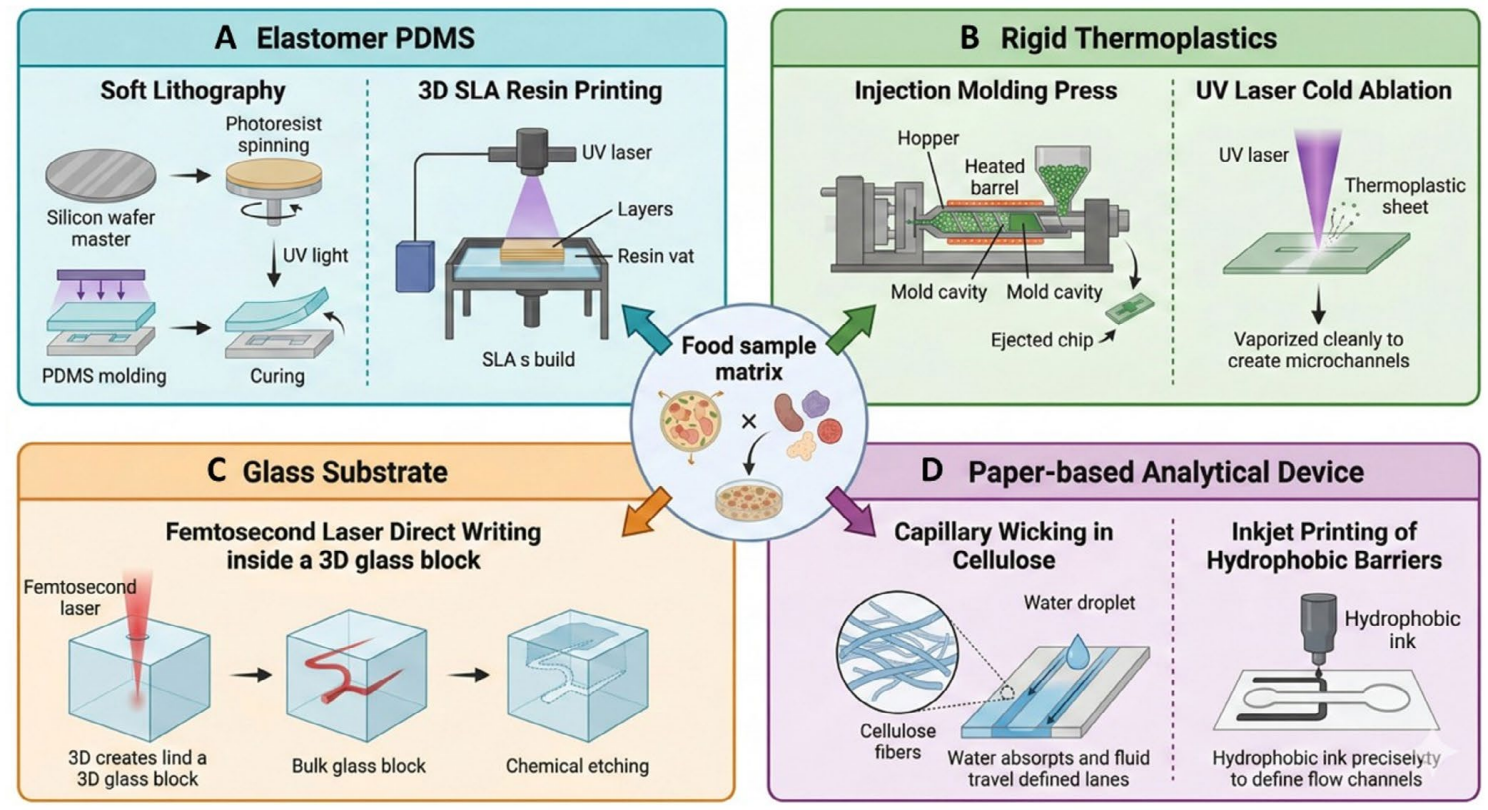
Diverse substrate materials and microfabrication methodologies for foodborne pathogen detection platforms. (A) Elastomeric PDMS devices fabricated via soft lithography and 3D SLA resin printing. (B) Rigid thermoplastics (e.g., PMMA, COC) utilized for high‐throughput commercial mass production. (C) Glass substrates exhibiting optical transparency and chemical resistance. (D) Paper‐based analytical devices (μPADs) demonstrating a low‐cost, pump‐free paradigm. (Some visual elements were generated with the assistance of Nano Banana and were incorporated by the authors into the complete figure using Microsoft PowerPoint. The completed figure was conceived, assembled, and scientifically reviewed by the authors. No third‐party copyrighted images, icons, templates, or other protected visual materials were used.)

### 3.1 Elastomer‐Based Platforms: PDMS And Fabrication Methodologies

PDMS remains the quintessential elastomeric substrate in microfluidic engineering, heavily overshadowing alternative elastomers like thermoplastic elastomers (e.g., SEBS, polyurethane) and hydrogels. Synthesized via the curing of a siloxane prepolymer with a crosslinking agent, PDMS offers exceptional optical transparency, high gas permeability, mechanical elasticity, and the capacity for reversible conformal sealing. However, its widespread deployment in commercial biological assays is severely impeded by critical material limitations that directly compromise diagnostic reliability. First, the inherent porosity and hydrophobicity of the PDMS polymer network trigger the severe non‐specific adsorption and bulk partitioning of small hydrophobic molecules (e.g., toxins, diagnostic fluorophores, and target analytes). This depletion drastically reduces the effective sample concentration, skewing quantitative data and artificially degrading the LOD in sensitive food safety screening (Grindulis et al. 2025). Second, while contemporary chips are frequently functionalized with surface modifiers (e.g., oxygen plasma) to mitigate biofouling and improve the capillary flow of aqueous samples, PDMS exhibits rapid “hydrophobic recovery.” Low‐molecular‐weight uncrosslinked oligomers diffuse from the bulk elastomer to the surface, causing microchannels to revert to their native hydrophobic state within hours to days. This thermodynamic instability fundamentally undermines the long‐term shelf‐life and reproducibility of ready‐to‐use biosensors (Tsuzuki et al. 2022).

Conventionally, PDMS platforms are fabricated utilizing soft lithography, a highly precise technique pioneered by Whitesides and colleagues. This process relies on replica molding against a master template, typically a silicon wafer patterned with an ultraviolet (UV)‐exposed photoresist (e.g., SU‐8). While soft lithography provides unparalleled nanoscale resolution, it remains a labor‐intensive, cleanroom‐dependent batch process. The geometric limitations and tedious multi‐layer alignment prevent the reliable mass‐manufacturing required to transition from laboratory prototypes to standardized point‐of‐care (POC) commercial products, posing an insurmountable bottleneck for large‐scale commercialization (Volpatti and Yetisen 2014). Soft‐lithography workflows can complicate scale‐up of a specific PDMS device; manufacturability, shelf life, and performance in food matrices require device‐level validation (Lu et al. 2024).

To circumvent these manufacturing bottlenecks and geometric limitations, high‐resolution 3D printing—particularly stereolithography (SLA)—has emerged as a transformative alternative. Operating via the layer‐by‐layer selective laser polymerization of liquid photopolymer resins, SLA facilitates the rapid prototyping of complex, truly three‐dimensional internal architectures, such as chaotic micromixers, that cannot be produced through planar molding. While historical SLA applications focused heavily on medical implants, recent advancements have redirected this technology toward food safety. Modern SLA is now extensively employed either to print rapid, low‐cost master molds for PDMS casting or to directly fabricate functionalized microneedle biosensor arrays, enabling the rapid, extraction‐free sampling and real‐time detection of contaminants directly from solid food matrices.

The advent of high‐resolution 3D printing has fundamentally transitioned microfluidic fabrication from planar soft lithography to genuine three‐dimensional architectures, enabling the seamless integration of sophisticated optical nanostructures directly into the fluidic network. This geometrical freedom is particularly crucial for multiplexed foodborne pathogen screening, where complex valving, micromixing, and sample splitting are imperative. A compelling demonstration of this capability was recently reported by Feng et al. (Feng et al. 2024), who developed a 3D‐printed microfluidic chip integrated with a highly sensitive aptamer‐based nanointerferometer. Designed iteratively via CAD and fabricated using advanced resin‐based stereolithography (SLA), the monolithic chip incorporates droplet inlets, precision fluidic flow channels, check valves, and spatially isolated optical sensing regions. By monitoring subtle optical signal shifts—specifically, the variation in interference fringe patterns induced by target‐aptamer binding events—via a compact, portable spectrometer, this 3D‐printed platform achieved the simultaneous, label‐free quantification of four critical foodborne pathogens: *Listeria monocytogenes* , *Escherichia coli* , *Salmonella typhimurium* , and *Staphylococcus aureus* . Such localized integration of optical nanostructures within a 3D‐printed robust polymeric matrix fundamentally circumvents the tedious multi‐layer alignment issues prevalent in traditional PDMS‐glass assemblies, drastically reducing both production costs and diagnostic turnaround times.

### 3.2 Thermoplastic‐Based Chips

Rigid thermoplastics have emerged as the cornerstone for the commercial translation of microfluidic devices, effectively categorized into three tiers: general‐purpose substrates like polymethyl methacrylate (PMMA) and polycarbonate (PC), low‐cost alternatives like polystyrene (PS), and high‐performance polymers including cyclic olefin copolymer (COC) and cyclic olefin polymer (COP). PMMA and PC offer robust mechanical strength, low protein adsorption, and straightforward surface functionalization. PS provides extreme cost‐efficiency and biocompatibility, making it a staple for mass‐produced cell culture and analytical platforms. Conversely, COC and COP represent the gold standard for optical‐based biosensing; they boast exceptional UV–visible transparency, negligible autofluorescence, formidable chemical inertness against aggressive solvents, and high dimensional stability. For foodborne pathogen detection, this inherent material robustness and excellent optical clarity are paramount, as they ensure high compatibility with portable, fully integrated diagnostic cartridges capable of processing complex food matrices in resource‐limited field settings (Liao et al. 2023; Lu et al. 2024). COC and COP may support scalable optical cartridge fabrication, but material selection alone does not establish field readiness. Each integrated device requires testing for manufacturing reproducibility and robustness under its intended use conditions (Zhang, Ma, et al. 2025; Zhang, Zhao, et al. 2025).

To transition these materials from prototyping to industrial‐scale production, researchers have heavily optimized high‐throughput manufacturing paradigms. Injection molding stands as the premier method for commercial mass production. Recent process optimizations have enabled the fabrication of PMMA microchannels with cycle times under 45 s, while PS‐based high‐throughput platforms have been successfully molded at high clamping forces in just 15 s to support high‐density single‐cell analysis. Furthermore, injection molding has been leveraged to imprint nanoscale features, such as embedded gold‐capped nanocones in COC substrates, tailored specifically for surface‐enhanced Raman scattering (SERS) applications. For food pathogen chip detection, this unparalleled mass production advantage means that highly reproducible, low‐cost disposable test cartridges can be manufactured at the scale required for routine, high‐throughput screening across the global food supply chain, thereby eliminating cross‐contamination risks (Fiorini and Chiu 2005; Han et al. 2026).

Beyond traditional injection molding, ultrasonic‐assisted hot embossing has gained significant traction as a rapid, energy‐efficient alternative. By applying ultrasonic vibrations (via a sonotrode) to substrates like PC or PMMA, localized interfacial friction rapidly heats the polymer above its glass transition temperature. This dynamic facilitates precise micropattern replication in a matter of seconds, circumventing the prolonged thermal cycling required by conventional hot embossing. For food pathogen chip detection, this rapid, localized, and solvent‐free sealing ensures the structural robustness of the chip while perfectly preserving the bioactivity of pre‐loaded, temperature‐sensitive recognition elements (e.g., antibodies or aptamers) critical for on‐site diagnostic accuracy (Schomburg et al. 2011; Zhu et al. 2021).

Finally, laser ablation has matured as a highly versatile, mask‐less technique for direct channel engraving. While earlier approaches relied heavily on continuous or pulsed CO2 lasers—which often left Gaussian‐like profiles and thermal degradation artifacts (e.g., melting and decomposition) on COP and PMMA substrates—contemporary methodologies increasingly adopt ultraviolet (UV) and femtosecond (fs) lasers. These advanced laser systems mitigate thermal damage through “cold ablation,” enabling the ultra‐precise, rapid fabrication of complex microfluidic networks with sub‐micron surface roughness, thereby matching the quality of precision micromilling while drastically reducing production time. For food pathogen chip detection, this highly agile, mask‐less customization means researchers can rapidly iterate and deploy complex fluidic architectures specifically tailored to isolate trace pathogens from highly heterogeneous and viscous food samples (Hu et al. 2020; Ren et al. 2024).

### 3.3 Glass‐Based Chips

Occupying a strategic position in high‐grade biochemical analysis, glass‐based microfluidic platforms are broadly classified into silicate glasses (e.g., soda‐lime, borosilicate, and fused silica) and photosensitive glasses (Aralekallu et al. 2023). These inorganic substrates are renowned for their unparalleled optical transparency, formidable mechanical strength, and absolute chemical inertness against harsh organic solvents. Furthermore, their inherently hydrophilic surfaces provide abundant silanol groups, facilitating highly stable, covalent surface functionalizations. Photosensitive glasses uniquely permit direct photolithographic patterning and anisotropic chemical etching to generate intricate 3D architectures. Historically relying on cleanroom‐dependent photolithography, glass microfabrication has recently adopted dry film photoresists (DFR) as cost‐effective, easily laminable pattern‐transfer masks for rapid wet etching (Zhang et al. 2015). Moreover, this paradigm has been profoundly disrupted by femtosecond laser direct writing (FsLDW) and laser‐induced chemical etching (FLICE). This mask‐less, ultrafast laser processing enables the high‐precision engraving of true 3D microchannels and superhydrophobic functional features directly within the glass volume, establishing a new frontier for high‐performance droplet generation and pathogenic screening (Liao et al. 2025).

### 3.4 Paper‐Based Microfluidic Chips

This category encompasses 2D paper chips, 3D paper architectures, and flexible textile‐based microfluidics, fundamentally united by their low‐cost, pump‐free nature and profound suitability for resource‐limited environments (García‐Azuma et al. 2024). Fabricated predominantly from chromatographic cellulose, 2D microfluidic paper‐based analytical devices (μPADs) exploit spontaneous capillary wicking to drive fluid flow, entirely circumventing the need for external micropumps. They offer compelling advantages for PON diagnostics, including extreme cost‐efficiency, a high‐contrast white background for colorimetric readouts, and safe disposal via incineration. However, while these paper‐based platforms are promising for rapid qualitative screening, they are not always suitable for precise quantification; environmental humidity, flow variability, poor reproducibility, and limited sensitivity remain major constraints (Sinha et al. 2022). To achieve higher integration, 3D μPADs are constructed by stacking or folding multiple 2D layers; this creates complex spatial fluidic networks, prevents signal crosstalk, and facilitates multiplexed pathogen detection. Concurrently, textile‐based platforms utilizing cotton or blended threads provide superior mechanical flexibility and structural durability for harsh‐environment sensing. The main fabrication methodologies contain wax and piezoelectric inkjet printing. Historically, wax printing dominated μPAD prototyping; it rapidly deposited hydrophobic wax that permeated the porous cellulose matrix via thermal reflow to form three‐dimensional fluidic walls (Carrilho et al. 2009). While cost‐effective, the discontinuation of commercial solid‐ink printers and inherent issues with boundary blurring—caused by isotropic wax spreading during heating—have necessitated new approaches (Ruiz et al. 2022). Consequently, contemporary microfluidic manufacturing increasingly favors piezoelectric inkjet printing. Utilizing precise picoliter droplet control, inkjet techniques deposit custom hydrophobic polymers directly onto substrates without physical stencils, substantially improving spatial resolution and boundary fidelity (Abe et al. 2008). These high‐precision, mask‐less methodologies perfectly align with the rigorous demands of multiplexed chemical and biological assays in modern food safety monitoring. Ultimately, modern microfluidic substrate selection is governed by distinct application paradigms: glass for high‐fidelity optics, polymer for scalability, and paper for low‐cost field deployment (Deb et al. 2026).

Despite their accessibility, μPADs frequently exhibit diminished diagnostic accuracy when confronted with lipid‐rich food emulsions; therefore, progression of paper‐based devices toward deployable commercial status may benefit from orthogonal pre‐filtration membranes that reduce matrix interferents before capillary wicking; reliability under diverse lipid‐rich food emulsions still requires validation (Wu et al. 2024).

## 4 Integrated Biosensing Modalities in Microfluidic Platforms

Beyond the intricacies of pure signal transduction, the precise pre‐analytical separation of trace pathogenic bacteria from heterogeneous, opaque food matrices (e.g., unpasteurized milk, raw meat extracts) remains the fundamental bottleneck in translating food safety engineering to the field. Advanced microfluidic platforms have recently pioneered continuous‐flow sorting mechanisms utilizing label‐free hydrodynamic and viscoelastic forces. By meticulously tuning the viscoelastic properties of the carrier fluid and leveraging inertial migration phenomena within specifically tailored microchannel geometries, researchers can achieve the size‐dependent deterministic lateral displacement of bacterial cells. Zhang et al. demonstrated shape‐based, label‐free separation of drug‐treated *Escherichia coli* using viscoelastic microfluidics (T. Zhang, Huang, et al. 2022; Zhang, Liu, et al. 2022). Following successful isolation, droplet digital microfluidics (ddMF) enables the absolute quantification of individual pathogens without reliance on standard calibration curves. By encapsulating single bacterial cells or their extracted nucleic acids into thousands of independent picoliter‐sized water‐in‐oil droplets, target concentration becomes a function of Poisson statistics. Recent integrations of droplet generation with CRISPR‐Cas13a or loop‐mediated isothermal amplification (LAMP) have achieved nucleic acid amplification‐free, absolute quantification of trace *E. coli* and SARS‐CoV‐2 variants, illustrating the potential of droplet microfluidics for low‐abundance and emerging drug‐resistant foodborne pathogens, while broader food‐matrix validation and workflow integration remain necessary.

The integration of robust transduction mechanisms is pivotal for the analytical performance of microfluidic devices, with optical, electrochemical, and plasmonic biosensors constituting the primary modalities (As shown in Table 1). Optical detection—encompassing fluorescence, chemiluminescence, and colorimetry—delivers exceptional spatial resolution, non‐destructive analysis, and multiplexing capabilities, making it indispensable for nucleic acid quantification and single‐cell profiling. Conversely, electrochemical sensing translates biological recognition events into measurable electrical signals, offering distinct advantages in miniaturization, rapid response times, and cost efficiency. This modality is particularly resilient to optical interferences in complex food matrices, establishing it as a cornerstone for POC pathogen and toxin screening. As a label‐free electrochemical subset, impedance‐based detection enables the real‐time, non‐invasive monitoring of cellular dynamics and bacterial proliferation, even within highly opaque media. Furthermore, surface plasmon resonance (SPR) and localized SPR (LSPR) exploit the extreme sensitivity of metal nanostructures to local refractive index fluctuations. These plasmonic techniques facilitate the label‐free, real‐time kinetic analysis of biomolecular interactions, with LSPR providing enhanced spatial resolution for single‐molecule and spatial receptor imaging. Given the inherent disparities in sensitivity, matrix compatibility, and operational complexity among these transduction mechanisms, selecting an optimal “fit‐for‐purpose” detection strategy is imperative. The subsequent sections will detail the specific application of these integrated modalities in diverse target screening.

**TABLE 1:** Selected microfluidic biosensors for foodborne pathogens. Reported limits vary by matrix, unit, pretreatment, and enrichment and are not directly comparable.

Analyte	Food matrices	Chip type	Detection method	Limit of detection	Ref
*Salmonella*	Food culture	Lateral flow microfluidic biosensor	Colorimetric and photothermal speckle imaging	10^4^ CFU/mL	(Blackshare et al. 2026)
*Escherichia coli* O157:H7	Orange juice and milk	Dual‐RCA aptamer microfluidic platform	Aptamer capture with dual‐RCA fluorescence readout	80 cells/mL	(Jiang et al. 2020)
*Salmonella enterica*	Lettuce, chicken breast, and milk	Polyethersulfone (PES)‐based paper microfluidic device	RPA with lateral‐flow readout	6 CFU/g in lettuce after 6 h enrichment	(Chen et al. 2023)
*Escherichia coli* O157:H7	Potable water	Monoclonal antibody‐based non‐Faradaic immunoassay	Electrochemical Impedance Spectroscopy	1 CFU/mL	(Mishra et al. 2024)
*Salmonella choleraesuis*	Undiluted milk	Microfluidic device with top‐bottom IDE pairs	Electrochemical Impedance Spectroscopy	Calculated 2 CFU/mL; 50 min total	(Police Patil et al. 2025)
*Escherichia coli*	Environmental and food safety samples	Pencil‐on‐paper microfluidic platform	Electrochemical and optical detection	2.7 × 10^2^ CFU/mL	(Soysaldı et al. 2024)
*Escherichia coli*	Water	CIP‐functionalized stainless steel microwires in PDMS	Electrochemical Impedance Spectroscopy	2 × 10^2^ CFU/mL	(Akhtarian et al. 2024)
*Salmonella* and *Vibrio* species	Food matrices	CsPbBr_3_ perovskite quantum dots platform	Fluorescence detection	10 CFU/mL	(Mohammad et al. 2026)
*Salmonella typhimurium*	Complex anaerobic food homogenates	Self‐oxygenating catalytic nanozyme biosensor	Colorimetric and fluorescence detection	10^3^ CFU/mL	(Deng et al. 2026)
*Vibrio parahaemolyticus*	Food samples	CuO_2_ nanodot‐encapsulated MOF microfluidic chip	Dual‐mode colorimetric‐electrochemical	30 CFU/mL	(Wang et al. 2023)

### 4.1 Optical Detection: Colorimetry

Colorimetry is a foundational analytical technique governed by the Beer–Lambert law, which quantifies the linear relationship between the optical absorbance of a chromophore and its concentration (Guo et al. 2021). By measuring target‐induced variations in optical absorption—often resulting from specific electronic transitions or LSPR phenomena—this methodology achieves precise qualitative and quantitative molecular detection. This methodology primarily encompasses two broad reaction paradigms: enzyme‐catalyzed chromogenic reactions and nanoparticle aggregation.

The first paradigm involves enzyme‐catalyzed chromogenic reactions. Alkaline phosphatase (ALP), a quintessential enzymatic label, is a homodimeric metalloenzyme whose catalytic core obligately requires two zinc ions (Zn^2+^) and one magnesium ion (Mg^2+^) to maintain optimal physiological activity. Mammalian ALPs are classified into four principal isoenzymes: germ cell, intestinal, placental (PLAP), and tissue‐nonspecific alkaline phosphatase. Crucially, ALP isoenzymes serve as robust prognostic biomarkers for various malignancies; for instance, PLAP is frequently overexpressed in colorectal adenocarcinomas and has recently emerged as a highly specific target for chimeric antigen receptor (CAR) T‐cell therapies. In clinical diagnostics, ALP activity is routinely quantified in diverse sample types, including complex human serum, plasma, and tissue homogenates. Recent advancements have established rigorous sensitivity boundaries, achieving limits of detection (LOD) as low as 1.25–1.75 mU/mL in solution‐based clinical serum matrices (Hu et al. 2017), while paper‐based formats maintain sufficient sensitivity to rapidly detect abnormal physiological thresholds above 30 mU/mL (Gao et al. 2014).

Colorimetric assays are intrinsically rapid, cost‐effective, and portable. These attributes make them highly adaptable for POCT, particularly when integrated with μPADs. Recently, Li, Dou, et al. (2024); Li, Wang, et al. (2024) engineered a programmable smartphone‐assisted μPAD utilizing a bioenzyme‐nanozyme cascade for the simultaneous colorimetric detection of multiple enzymes, including ALP. By employing cobalt‐doped mesoporous cerium oxide to amplify the signal, the platform realized limits of detection orders of magnitude lower than conventional paper‐based methods (K. Li, Dou, et al. 2024; Li, Wang, et al. 2024). Furthermore, colorimetric μPADs demonstrate exceptional utility in food safety monitoring. Broten et al. (2022) developed a rapid, genus‐specific μPAD for detecting on environmental surfaces, successfully detecting concentrations as low as 10^2^ CFU/coupon. Despite their extreme cost‐efficiency, equipment‐free operation, and pump‐free fluidics, paper‐based colorimetric sensors exhibit critical limitations. Their practical utility is frequently compromised by low intrinsic sensitivity, restricted quantitative analytical efficiency, and heterogeneous color formation hindered by inadequate interlayer attachment and the intrinsic structural heterogeneity of the cellulose matrix (Morbioli et al. 2017).

The second paradigm relies on nanoparticle aggregation. Integrating functionalized nanomaterials with microfluidics facilitates the multiplexed analysis of proteins, nucleic acids, and whole cells. Gold nanoparticles (AuNPs) are particularly favored for POCT due to their unique LSPR. In a foundational approach, Jiang and colleagues developed a highly sensitive colorimetric immunoassay targeting the HIV‐1 gp41 antigen. The assay utilizes copper‐mediated cross‐linking between functionalized AuNPs, inducing a distinct LSPR shift from red to blue, achieving a naked‐eye LOD of 150 ng/mL. However, the deployment of aggregation‐based assays in complex biological or food matrices is severely hindered by non‐specific matrix interference. High concentrations of heterogeneous biomolecules rapidly adsorb onto the nanomaterial surface, forming a “protein corona” that fundamentally alters nanoparticle physicochemical properties and precipitates non‐specific aggregation. This phenomenon severely distorts optical signals and generates false positives unless mitigating strategies, such as stringent surface passivation via PEGylation, are systematically implemented (Feng et al. 2021).

To systematically eradicate the limitations of subjective naked‐eye interpretation, background noise, and complex matrix interference, the convergence of μPADs with smartphone‐based imaging and Artificial Intelligence (AI) may provide a potential approach for quantitative POCT, with performance dependent on representative image datasets and validation across food matrices. Deep learning architectures, particularly Convolutional Neural Networks (CNNs) and the You Only Look Once (YOLO) object detection algorithm, are now routinely deployed to dynamically correct illumination biases, segment detection zones, and extract high‐fidelity colorimetric data. For instance, Blackshare et al. (2026) developed a portable biosensor for *Salmonella* utilizing lateral flow microfluidics combined with machine learning, minimizing coefficient of variation errors to negligible levels (Blackshare et al. 2026). Similarly, Zhu et al. (2025) introduced ‘TimePAD’, an innovative deep learning‐driven approach that unveils temporal sequence ELISA signals on a microfluidic platform. Instead of relying on a single end‐point image, TimePAD analyzes the kinetic evolution of color development, significantly improving predictive accuracy for trace analytes even in highly turbid food homogenates (Zhu et al. 2025). Ultimately, these AI‐augmented microfluidic systems can extend optical‐readout analysis by supporting image interpretation and signal classification; their practical advantages require prospective validation against conventional workflows before broad deployment can be inferred (Merkoçi 2025).

### 4.2 Optical Detection: Fluorescence

Rather than simply restating the textbook “absorption‐reemission” phenomenon, contemporary microfluidic fluorescence biosensing exploits the precise translation of biochemical recognition events into quantifiable photon emissions within confined microenvironments. By manipulating fluid dynamics at the microscale, these platforms maximize the signal‐to‐noise ratio and capture efficiency of complex biomolecular targets. The evolution of this analytical principle has transitioned from basic affinity‐based signal transduction to highly integrated architectures. Based on their signal generation mechanisms, these representative integrated designs can be broadly categorized into three distinct modalities: fluorescence alone, fluorescence coupled with isothermal amplification, and fluorescence synergized with CRISPR‐Cas systems.

In platforms utilizing fluorescence alone, detection relies primarily on affinity‐based bio‐recognition elements, such as aptamers. For instance, a dual‐RCA aptamer microfluidic platform has successfully isolated *Escherichia coli* O157:H7 from complex matrices like orange juice and milk, with a reported limit of 80 cells/mL (Jiang et al. 2020). While this modality excels in procedural simplicity and continuous flow screening, it inherently suffers from high background noise due to autofluorescence from sample matrices and microfluidic polymeric substrates. Matrix autofluorescence and polymer background can impair fluorescence readout in some foods, so analytical performance should be verified after sample preparation and with matrix‐matched controls. Furthermore, its equipment dependency remains high, often necessitating bulky auxiliary equipment like high‐power lasers or photomultiplier tubes (PMTs). Consequently, featuring moderate readout complexity that requires standard calibration curves, fluorescence alone is best suited for rapid, front‐end screening in centralized laboratories rather than definitive trace‐level diagnostics.

To achieve absolute quantification and overcome the sensitivity limits of pure affinity assays, fluorescence readouts are powerfully synergized with isothermal amplification. A representative state‐of‐the‐art design is the digital‐to‐droplet microfluidic device developed by Xie et al. (2024), which executes an integrated multiplex digital LAMP assay. This platform automates droplet generation and incubation, enabling the simultaneous quantification of multiple uropathogens within 75 min. This modality's primary strength is its capability to provide standard‐free, absolute quantification. However, its weaknesses include a moderate‐to‐high equipment dependency—requiring automated droplet generators or complex microfluidic circuitry—and high readout complexity demanding sophisticated statistical image analysis for droplet counting. Therefore, this technology presents a highly realistic application scenario in acute clinical settings requiring precise multiplexed pathogen profiling.

Finally, the integration of CRISPR‐Cas effectors with microfluidics has enabled advanced laboratory prototypes that use the collateral trans‐cleavage of Cas12a/13a to degrade fluorophore‐quencher reporters upon specific target recognition; their performance may nevertheless vary across samples and workflows. Chen et al. (2023) engineered a polyethersulfone (PES)‐based paper microfluidic device that seamlessly integrates DNA extraction, Recombinase Polymerase Amplification (RPA), and CRISPR/Cas12a‐mediated fluorescence detection (Chen et al. 2023). By preventing premature degradation via circular DNA reporters, advanced systems can exponentially enhance the fluorescence signal, pushing the LOD down to the femtomolar range within just 35 min (Liu et al. 2025). However, while CRISPR‐assisted modalities can provide very low analytical limits of detection in controlled settings, their commercial viability remains constrained by biochemical buffer incompatibilities and susceptibility to matrix biofouling when exposed to unprocessed food samples. High lipid content in milk or protein debris in meat extracts can physically occlude the CRISPR‐Cas complex, severely inhibiting collateral trans‐cleavage kinetics (Feng, Shi, et al. 2025; Feng, Zhao, et al. 2025). To circumvent this, the development of orthogonal CRISPR assays—utilizing multi‐gRNA panels and orthogonal Cas proteins (e.g., pairing Cas12a with Cas13a) within spatially isolated microfluidic compartments—is a proposed engineering option whose benefit requires experimental validation. This orthogonal architecture may support simultaneous multiplexed pathogen detection while reducing signal crosstalk; any reduction in matrix‐induced false positives requires validation in integrated devices using authentic samples (Li et al. 2026). Systematically, the CRISPR‐assisted modality exhibits ultra‐low background noise due to its dual‐recognition mechanism, exceptionally low equipment dependency utilizing portable heaters or paper substrates, and minimal readout complexity yielding direct visual or smartphone‐readable thresholds. Although limited by biochemical buffer mismatches requiring spatial compartmentalization, its high analytical specificity makes it a promising candidate for decentralized, point‐of‐need food safety surveillance and field diagnostics, provided that matrix compatibility and operational robustness are demonstrated.

### 4.3 Electrochemical Sensing

The synergistic integration of electrochemical sensing with microfluidic technology capitalizes on the intrinsic advantages of both paradigms, yielding highly miniaturized, automated, and high‐throughput diagnostic platforms. Electrochemical sensors are fundamentally characterized by their rapid response kinetics, compact architectural footprint, low cost of fabrication, and seamless capacity for miniaturization, representing robust POC alternatives for environmental and food safety screening (J. Zhang, Ma, et al. 2025; Zhang, Zhao, et al. 2025). Based on their distinct transduction mechanisms, contemporary electrochemical biosensors for pathogen detection are primarily classified into two standardized analytical modalities: amperometric/voltammetric and impedimetric sensing (Fukana et al. 2025). Crucially, unlike optical techniques, electrochemical modalities are inherently insensitive to sample opacity and autofluorescence, rendering them exceptionally well‐suited to bypass optical matrix interference prevalent in highly turbid food homogenates (Cunha et al. 2025).

#### 4.3.1 Amperometric and Voltammetric Detection

Amperometric and voltammetric biosensors function by measuring the Faradaic current generated from the electrochemical oxidation or reduction of electroactive species at the electrode‐solution interface (Fdez‐Sanromán et al. 2025). In amperometry, the current is continuously monitored under a constant applied potential, which affords excellent sensitivity and allows for real‐time tracking of biological binding events. Conversely, voltammetric techniques measure the current response as a function of a continuously varied potential, establishing highly specific electrochemical redox profiles that enhance analytical selectivity (Manring et al. 2022).

While these current‐based modalities offer profound baseline sensitivity, they typically require the presence of electroactive labels, redox mediators, or specific enzymatic reactions to facilitate efficient electron transfer between the target pathogen and the electrode. To overcome these constraints and amplify signals, recent microfluidic devices have aggressively integrated advanced nanomaterials. For instance, microfluidic voltammetric sensors employing conductive composite materials, such as graphene oxide‐wrapped carboxylated multi‐walled carbon nanotubes (GO‐cMWCNTs), have achieved highly selective and sensitive detection of *Salmonella typhimurium* by accelerating charge transfer kinetics directly at the sensing interface (Zhang, Ma, et al. 2025; Zhang, Zhao, et al. 2025).

#### 4.3.2 Impedimetric Detection

In contrast to current‐based methodologies, impedimetric detection remains one of the most powerful label‐free approaches for bacterial sensing. The fundamental principle involves monitoring subtle variations in interfacial electrical impedance induced by the specific binding of target analytes (Furst and Francis 2019). These target‐receptor interactions perturb the electrical double layer or alter charge transfer kinetics, which are subsequently transduced into quantifiable electrical signals to achieve precise qualitative and quantitative analysis without the need for complex enzymatic labeling.

These measurements are typically executed using interdigitated microelectrode (IDE) arrays integrated directly into microfluidic channels. Compared to conventional macroscopic electrodes, IDEs offer profound analytical advantages: they possess a dramatically increased active surface‐to‐volume ratio, establish steady‐state mass transport rapidly, and require only microliter sample volumes. Furthermore, contemporary optimizations in IDE geometry mitigate edge effects and ensure a uniform electric field distribution, thereby yielding superior signal‐to‐noise ratios and enabling ultra‐sensitive, real‐time pathogen quantification.

Recently, advanced electrochemical impedance spectroscopy (EIS) platforms have enabled rapid detection of *Escherichia coli* O157:H7. Moving beyond early bulk‐electrode designs, modern portable non‐Faradaic EIS sensors utilizing specific monoclonal antibodies immobilized on microelectrodes have demonstrated exceptional sensitivity. These contemporary platforms can detect *E. coli* O157:H7 with a remarkable limit of detection (LOD) of just 1 CFU/mL within a 5‐min assay time (Mishra et al. 2024).

Police Patil et al. (2025) reported a microfluidic device with top‐bottom electrode pairs that combine positive dielectrophoretic concentration with impedimetric sensing of *Salmonella enterica* serovar Choleraesuis in spiked undiluted milk. The authors reported a calculated analytical detection limit of 2 CFU/mL and a total procedure time of 50 min. These results are promising under the specified test conditions, but the reported analytical limit should not be interpreted as performance across all food matrices or as field validation.

Early iterations of paper‐based impedance immunosensors for pathogen detection often suffered from inadequate sensitivity and high detection limits. To overcome these constraints, modern biosensor design integrates advanced nanomaterials. For example, a recent study introduced a highly sensitive paper‐based impedimetric platform utilizing magnetic core‐shell Fe_2_O_3_@CdSe/ZnS quantum dots. By exploiting EIS, this platform successfully quantified *E. coli* with a substantially improved LOD of 2.7 × 10^2^ CFU/mL and an analysis time of just 30 min, representing a robust point‐of‐care alternative for environmental and food safety screening (Soysaldı et al. 2024).

Furthermore, the utilization of synthetic bio‐recognition elements has significantly enhanced the environmental durability of microfluidic impedimetric platforms. Akhtarian et al. integrated cell‐imprinted polymer (CIP)‐functionalized stainless steel microwires into a PDMS microfluidic device for the label‐free detection of *E. coli* . The integrated sensor features parallel microchannels with a three‐electrode configuration, enabling simultaneous microfluidic control and real‐time EIS measurements. Following a 30‐min incubation, the specific binding of target bacteria to the CIP microwires induced a proportional increase in charge transfer resistance. By extracting the equivalent electrical circuits and analyzing the dose–response curve, the sensor demonstrated a robust dynamic range spanning from 10^2^ to 10^7^ CFU/mL, alongside a highly sensitive LOD of 2 × 10^2^ CFU/mL (Akhtarian et al. 2024). This architecture offers a highly sensitive, cost‐effective, and durable solution for the on‐site identification of waterborne pathogens.

#### 4.3.3 Horizontal Comparison and Practical Advantages

To elucidate the analytical capabilities of these emerging paradigms, a horizontal comparison of recent state‐of‐the‐art biosensors underscores the trade‐offs and synergies between transduction modalities. Current‐based approaches, such as the voltammetric microfluidic sensor utilizing GO‐cMWCNTs for *S. typhimurium* detection (Zhang, Ma, et al. 2025; Zhang, Zhao, et al. 2025), provide rapid and highly sensitive quantification through robust redox amplification. However, impedimetric platforms simplify the assay architecture by eliminating the need for electroactive labels. For instance, integrated microfluidic EIS systems can achieve an exceptional LOD of 1 CFU/mL within 5 min for *E. coli* O157:H7 using non‐Faradaic measurements (Mishra et al. 2024), and 2 CFU/mL within 50 min for *S. choleraesuis* when coupled with pDEP concentration (Police Patil et al. 2025). Meanwhile, cost‐effective alternative substrates, such as paper‐based EIS sensors leveraging Fe_2_O_3_@CdSe/ZnS quantum dots (Soysaldı et al. 2024) or microfluidic chips embedded with CIP microwires (Akhtarian et al. 2024), yield competitive LODs of 2.7 × 10^2^ CFU/mL and 2 × 10^2^ CFU/mL, respectively, within a 30‐min window.

The comparative analysis reveals that while amperometric and voltammetric sensors provide superior signal amplification via redox reactions, impedimetric sensors simplify the analytical workflow by enabling label‐free monitoring. Crucially, the integration of microfluidics with electrochemical sensing may mitigate some limitations of benchtop assays. By leveraging miniaturized geometries and paper‐based substrates, these contemporary platforms inherently reduce sample consumption, drive down fabrication costs, and enable extreme portability (Fukana et al. 2025). This paradigm shift firmly establishes electrochemical microfluidic sensors as a potential strategy for automated, point‐of‐need quantification of pathogens in resource‐limited and on‐site environments.

Smartphone‐connected electrochemical readers may support portable data collection and interpretation. However, the reported demonstrations should be distinguished from a fully integrated food‐testing system: stable calibration, performance in authentic matrices, user operation, and independent field validation remain to be shown for each device.

### 4.4 Surface Plasmon Resonance and Localized Surface Plasmon Resonance

SPR and LSPR are advanced, label‐free optical techniques that detect biomolecular interactions via refractive index shifts at the dielectric‐metal interface. While conventional SPR relies on continuous noble metal films, LSPR is confined to nanoscale metallic geometries, thereby circumventing the need for bulky prism‐coupling optics and rendering it highly amenable to extreme miniaturization. Synergizing these plasmonic phenomena with microfluidic networks profoundly enhances analytical performance by minimizing sample consumption, automating fluidic manipulation, and enabling high‐throughput multiplexing. Contemporary SPR imaging (SPRi) platforms integrate highly sophisticated microfluidic arrays. As highlighted by Escobedo and Brolo (2025) (Escobedo and Brolo 2025), modern optofluidic arrays drastically accelerate immunoreactions via active micro‐mixing and enable the real‐time quantification of biomarkers in complex matrices long before thermodynamic equilibrium is reached. Similarly, LSPR microfluidic platforms have achieved unprecedented sensitivities. Recently, Cetin and colleagues (2024) (Karaca Acari et al. 2024) developed an advanced optofluidic plasmonic biosensor for the rapid clinical diagnosis of Familial Mediterranean Fever, achieving label‐free, ultra‐sensitive detection of disease‐specific biomarkers directly from patient biofluids, significantly outperforming conventional laboratory instruments.

To achieve single‐molecule sensitivity and multiplexed fingerprinting, Surface‐Enhanced Raman Scattering (SERS) can be seamlessly integrated into these microfluidic architectures. By confining plasmonic nanostructures within continuous‐flow channels, the stochastic nature of static SERS hotspots is mitigated, yielding highly reproducible kinetic measurements. Recent breakthroughs substitute standalone noble metals with engineered hybrid nanostructures to exponentially amplify the electromagnetic field. For example, the incorporation of Copper‐Silver (Cu‐Ag) hybrid nanostructures into paper‐free plasmonic platforms generates ultra‐dense electromagnetic hotspots, significantly suppressing background interference and enhancing the LSPR signal (Rishabchaturvedi et al. 2026). This enables the rapid detection of highly toxic mycotoxins and pathogenic whole cells directly from agricultural wash water with unprecedented stability. Concurrently, the application of Cesium lead bromide (CsPbBr_3_) perovskite quantum dots (PQDs) has redefined fluorescence‐based microfluidic sensing. Utilizing remarkably high photoluminescence quantum yields and narrow emission spectra, mechanisms such as fluorescence resonance energy transfer (FRET) allow these PQD platforms to achieve detection limits as low as 10 CFU/mL for *Salmonella* and *Vibrio* species in complex food matrices, effectively bypassing typical biological autofluorescence (Mohammad et al. 2026).

Despite these remarkable analytical capabilities, the translation of such high‐sensitivity optical platforms to real‐world food detection faces critical limitations. The practical deployment of SPR, LSPR, and SERS biosensors is frequently hindered by high instrument complexity and the strict optical alignment required for stable signal transduction (Bryan et al. 2024). Furthermore, the fabrication of sophisticated nanostructured arrays imposes prohibitive costs that limit commercial scalability. Crucially, when applied to real food matrices, these sensors suffer from severe biofouling—driven by the non‐specific adsorption of complex proteins and lipids—which critically degrades target recognition, analytical reproducibility, and overall sensor lifespan (Xia et al. 2024).

Although LSPR‐ and SERS‐based platforms have demonstrated high analytical sensitivity under controlled laboratory conditions, their translation to complex food matrices remains challenging because of optical scattering, nonspecific adsorption, and matrix‐associated signal interference. Future plasmonic biosensing systems may benefit from complementary readouts, such as SERS and impedimetric sensing. The advantages of these multimodal approaches require comparison with single‐mode systems using authentic food samples and standardized workflows (Madadelahi et al. 2025).

### 4.5 Hybrid and Emerging Directions

Quartz crystal microbalance (QCM) sensors detect changes in resonant frequency caused by interfacial mass loading and can provide label‐free measurements. QCM is an established analytical principle, including in non‐food immunosensing applications (Kim et al. 2025). That history does not establish the readiness of a miniaturized QCM platform for foodborne pathogen testing. Such a device would require evidence on matrix fouling, environmental drift, sample handling, and performance outside the laboratory before claims about field use could be made.

Conversely, chemiluminescence‐based detection capitalizes on photon emission triggered by highly exergonic chemical reactions. Because this modality circumvents the need for an external optical excitation source, it inherently eliminates background scattering and autofluorescence, yielding exceptional signal‐to‐noise ratios. Recent breakthroughs have successfully integrated chemiluminescent reactions with digital microfluidics (DMF) and sophisticated microwell arrays, enabling the automated, ultra‐sensitive quantification of biomarkers directly at the point of care. Despite these advancements, integrated microfluidic chemiluminescence platforms require device‐specific validation, necessitating further optimization for mass manufacturability and reagent stability.

### 4.6 Orthogonal Sensing Architectures for Matrix Biofouling Mitigation

Matrix interference is a recurring barrier to translating laboratory biosensors into food‐testing workflows. The extent of interference depends on the sample and sensor design, so it should be evaluated with authentic foods and suitable controls. Orthogonal sensing is one potential engineering response, but evidence of improved accuracy over a single readout must be demonstrated for each integrated device (Wu et al. 2024).

Orthogonal biosensing combines distinct recognition mechanisms or readouts within one assay. For example, an optical and an impedimetric channel might respond differently to some interferents. Their coexistence, however, does not provide intrinsic cross‐validation: shared sample preparation, non‐specific binding, or correlated matrix effects can bias both channels. Demonstrated studies should therefore be distinguished from proposed architectures, and any claim of error reduction should be supported by paired‐channel controls, relevant food matrices, and comparison with a single‐mode system (Zhang, Huang, et al. 2022; Zhang, Liu, et al. 2022).

### 4.7 Qualitative Assessment of Translational Readiness

Technology readiness level (TRL) terminology is used here only as a heuristic for discussing translation. This narrative review did not apply a formal, system‐by‐system TRL assessment, and it does not assign a definitive TRL to a sensing modality or material class. Instead, the discussion distinguishes laboratory proof of concept, validation of an integrated prototype in authentic foods, and evaluation of a specific system in its intended operating environment.

For foodborne‐pathogen testing, prototype validation should include target recovery, matrix effects, total workflow time, repeatability, and stability in authentic foods. Later‐stage evidence would additionally require reproducible manufacturing, independent or multi‐site testing, field operation, and relevant regulatory evaluation. Table 2 summarizes these evidence stages as a qualitative guide; placement of a particular device requires device‐specific data rather than an assumption based on its substrate or readout.

**TABLE 2:** Qualitative evidence stages for translation of foodborne‐pathogen biosensors.

Evidence stage	Development stage	Evidence required before translation	Illustrative system characteristics
Laboratory proof of concept	Basic research & proof of concept	Analytical function and controls demonstrated in defined laboratory samples.	Examples may include individual sensor components tested in buffer
Integrated prototype validation	Prototype validation (“Valley of Death”)	Complete workflow tested in authentic food matrices with recovery, repeatability, and interference data.	Examples may include integrated devices tested with food samples
Field evaluation and deployment	System deployment & commercialization	Specific integrated system independently tested under intended operating conditions.	Examples require documented manufacturing and field performance

AI methods can assist the interpretation of complex biosensor signals, including drift correction and classification, when suitable datasets are available (Akkaş et al. 2025). The cited literature does not establish a common readiness level for AI‐assisted foodborne‐pathogen biosensors. Claims of improved diagnostic performance should be tied to a defined dataset, a comparator, representative food matrices, and independent validation. Explainable models may aid interpretation, but transparency alone does not establish robustness or regulatory acceptance.

The convergence of these transduction mechanisms with microfluidic networks fundamentally redefines the analytical landscape. Microfluidics empowers the deterministic manipulation of pico‐ to nanoliter volumes, which drastically curtails reagent consumption and accelerates mass‐transport‐limited reaction kinetics. Additionally, the immense surface‐to‐volume ratio characteristic of microchannels maximizes target capture efficiency. By automating complex sample preparation alongside integrated detection, these miniaturized platforms provide the essential infrastructure for deploying robust, high‐throughput diagnostic devices in resource‐limited environments.

The analytical sensitivity and environmental resilience of microfluidic biosensors are increasingly being augmented through the incorporation of multifunctional nanomaterials, specifically advanced catalytic nanozymes and Metal–Organic Frameworks (MOFs). While showing immense potential for signal amplification, these multifunctional nanomaterials still require device‐specific validation, requiring scalable synthesis pathways to ensure batch‐to‐batch reproducibility (Kidanemariam and Cho 2025). Nanozymes—inorganic nanomaterials exhibiting intrinsic enzyme‐like catalytic kinetics (e.g., peroxidase, oxidase, or catalase‐like activities)—offer remarkable stability against the harsh pH, salinity, and thermal conditions typical of food processing environments, overcoming the inherent fragility of natural biological enzymes. For the ultrasensitive detection of *Salmonella typhimurium* , Deng et al. (2026) engineered a highly innovative self‐oxygenating catalytic nanozyme‐based biosensor (Deng et al. 2026). This autonomous system effectively decomposes endogenous hydrogen peroxide to generate localized oxygen, thereby driving robust chromogenic or fluorescent oxidation reactions even within highly complex, anaerobic food homogenates, with reported catalytic performance under the study conditions. Furthermore, the extraordinary surface‐area‐to‐volume ratio and tunable porosity of MOFs facilitate the massive loading of signal probes and bio‐recognition elements (e.g., aptamers or antibodies). A recent state‐of‐the‐art dual‐mode colorimetric‐electrochemical microfluidic biosensor utilized CuO_2_ nanodots encapsulated within an MOF nanozyme framework to detect *Vibrio parahaemolyticus* (Wang et al. 2023). The MOF structure not only accelerated electron transfer at the microelectrode interface—drastically enhancing the electrochemical impedance response—but also catalyzed a secondary naked‐eye colorimetric readout. This dual‐mode study demonstrates complementary laboratory readouts; performance in authentic food matrices and field settings requires additional validation.

## 5 Summary and Outlook

Microfluidic biosensors have combined sample handling with amplification, immunoassays, and optical or electrochemical readouts to detect foodborne pathogens in selected experimental settings. Centrifugal, continuous‐flow, and paper‐based architectures offer different trade‐offs in pumping, fabrication, integration, and user operation; no one format can be identified as universally preferable from the studies reviewed here. The main translational challenge is to maintain analytical performance throughout a complete workflow using authentic foods. Future comparisons should report the original sample matrix, pretreatment, enrichment, measurement unit, assay time, and independent validation rather than ranking platforms on detection limit alone.

The first priority is to measure how sample preparation and matrix effects influence complete sample‐to‐answer performance. Orthogonal readouts and CRISPR‐assisted detection are candidates for comparative testing, not automatic safeguards against false results. Such studies should use appropriate controls, authentic and heterogeneous foods, and predefined performance criteria. Once analytical robustness is established, inter‐laboratory testing, reagent stability, usability, manufacturing reproducibility, and cost should guide subsequent development toward field use and regulatory evaluation.

In conclusion, the future trajectory of microfluidic food safety surveillance may benefit from a shift from endpoint detection toward integrated, orthogonal analytical systems, subject to comparative field validation. Next‐generation devices may use orthogonal methodologies—simultaneously targeting stable genomic DNA and functional proteins—to reduce false‐negative or false‐positive outcomes induced by thermal or chemical food processing, pending validation across processing conditions. Furthermore, the integration of data science must advance beyond generalized digital concepts; explainable machine learning (XAI) models may assist in the interpretation of orthogonal datasets and complex multiplexed readouts, but their ability to address dynamic matrix interference requires transparent training data and independent validation. Ultimately, the viability of these platforms depends on addressing sample pretreatment, device robustness, and the applicable regulatory requirements.

Future translation should follow a coordinated roadmap. First, scalable manufacturing requires reproducible thermoplastic or flexible‐material fabrication, standardized cartridge geometry, and stable reagent storage rather than one‐off prototype optimization (Feng, Shi, et al. 2025; Feng, Zhao, et al. 2025; R. Zhou, Ye, Ma, et al. 2025; R. Zhou, Ye, Zhang, et al. 2025; Wang et al. 2026). Second, standardized validation should use authentic food matrices, predefined analytical performance metrics, reference materials, and inter‐laboratory comparison before performance claims can be generalized. Third, commercialization requires sample‐to‐answer workflows that balance cost, usability, data governance, and regulatory quality assurance; AI‐assisted interpretation may support this process only when transparent, representative datasets and independent validation are available (X. Zhou et al. 2026).

## Author Contributions

**Wanjiao Mao:** writing – original draft, visualization, software, project administration, methodology, formal analysis, investigation, resources. **Li Zhang:** writing – review and editing, validation, project administration, investigation, funding acquisition, formal analysis, data curation, conceptualization, supervision.

## Funding

This work was supported by the Sichuan Science and Technology Program (Grant No. 2025ZNSFSC1614) and the Sichuan Medical Informatics Association (Grant No. 2025006) to LZ.

## Ethics Statement

The authors have nothing to report.

## Conflicts of Interest

The authors declare no conflicts of interest.

## Data Availability

Data sharing not applicable to this article as no datasets were generated or analysed during the current study.
